# An update of blood donor recruitment and retention in Hong Kong

**DOI:** 10.4103/0973-6247.42691

**Published:** 2008-07

**Authors:** C. K. Lee, J. Hong, A. T. F. Hung

**Affiliations:** *Department of Blood Collection and Donor Recruitment, Hong Kong Red Cross Blood Transfusion Service, Hong Kong, SAR, China*

**Keywords:** Blood donation, donor recruitment, donor retention

## Abstract

A healthy blood donor pool has to be well maintained in order to achieve self sufficiency in blood supply. Not only should new and young donors should be attracted and recruited into the pool so as to compensate the loss from drop out and deferred donors. At the same time, previous donors should be also actively retained to ensure they can come regularly. The status of donor recruitment and retention in Hong Kong is reviewed here to highlight the current difficulties in coping with increasing blood demand from an ageing population, stringent donor eligibility criteria and quality requirement in the blood collection. With a systemic analysis of the donation pattern, proposal is put forward to tackle the challenging problems.

## Background

**Figure 1:**
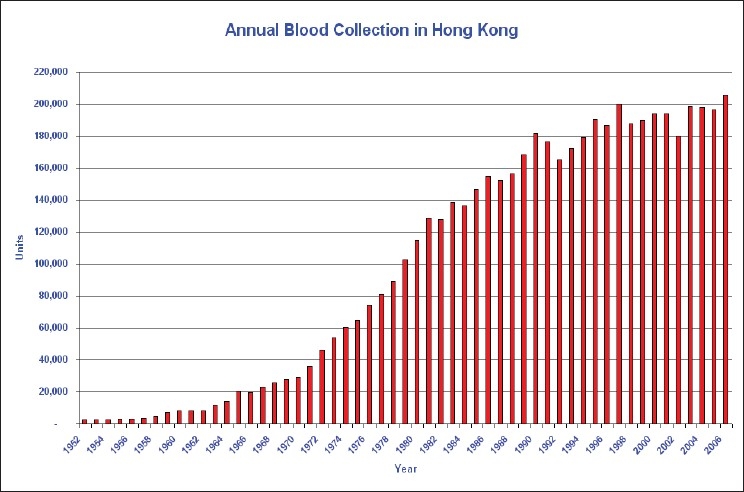
Annual blood collection in Hong Kong since 1952

**Figure 2:**
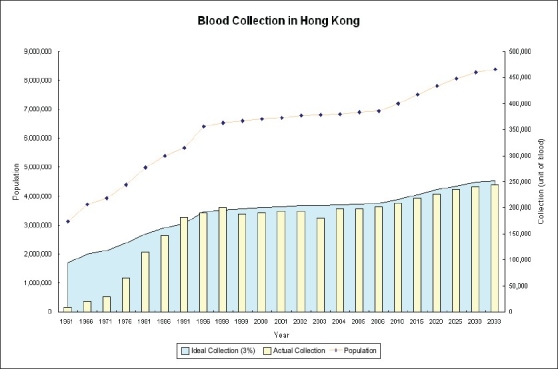
Estimation of blood collection required for the health care system in the next 25 years. Ideal collection is based on donation of 3% from the population

Blood donation programme in Hong Kong is run by the Hong Kong Red Cross Blood Transfusion Service (BTS). The BTS is responsible for the blood collection, processing, testing and distribution of blood to both public and private hospitals. It is being funded and managed by a public organization, Hospital Authority and is also accountable to the Hong Kong Red Cross who set up the service since 1952. All along the blood service was organized under the principle of voluntary non-remunerated blood donation. With the support from the public and more awareness, the annual blood collection is continued to increase over the past 50 years [Figure 1]. At present, the BTS collects about 200,000 units of whole blood for 6.9 million population. More importantly the concerted effort on promotion and education has changed the traditional mindset of the local Chinese to become supporters of the blood donation programme. Up to now, majority of the blood donors in Hong Kong are ethic Chinese and the BTS is able to maintain a relatively stable and adequate blood supply to patients requiring blood transfusion. However, with an ageing population as seen in many other developed countries, the BTS estimates that the blood demand will increase by 25% in next 25years [Figure 2]. By that time, it is forecasted that one quarter of the local citizens will be by the age of 65. Therefore, continued effort should be made to maintain the donor pool sufficient to meet the local blood demand.

### Problems in donor recruitment and retention

Donor recruitment and retention is not an easy task in blood service because of the dynamic nature of the socio-economic environment and the human factors involved. While the effectiveness of recruitment and retention is usually determined by the degree of adequacy in blood supply, some qualitative but important issues such as donor satisfaction and loyalty are frequently overlooked. It is well recognized that there always exists a group of otherwise healthy adult refuse to come forward for blood donation. On one hand many might think that blood donation is harmful to their health as in case of traditional Chinese belief. However, fears of blood and pain associated with donation are usually the commonest factors in their decision not to donate. At the moment, of the 200,000 donations each year, about 20% are collected from first time blood donors. About three quarter of the new donors are recruited from the school. Though it may be regarded as effective in the recruitment of the new donors, the long-term result of being regular donor is difficult to assess.

Similarly a bad personal experience or that learnt from peer is usually an excuse not to donate. Therefore, blood service should strive to provide better donor service or customer service and improved venepuncture skill to catch up with their expectation and minimize their willingness of not to donate. But they are often limited by scarce resources allocated to this area in comparison to that currently being used in laboratory testing for infectious diseases.

Recruitment of new donors serves two important purposes: (1) to compensate drop out or deferred donors and (2) to keep the pool expanding. However, in general there is a decreasing trend in the number of eligible first time donors as seen elsewhere. At the same time, an increasing ageing population limits the effectiveness of donor recruitment. Moreover, the increasingly stringent donor selection criteria employed in developed countries have resulted in a significant reduced proportion of eligible donors. It is not uncommonly to receive complaints from otherwise healthy regular donors being deferred for being traveled for a certain part of the world related to malaria risk. As a result, 10% or more deferral in prospective donors are often seen in developed countries. Therefore, extra effort should be put on to develop good recruitment strategies that are culturally sound and socially acceptable to the population concerned. Without such a mean to keep the blood donor pool growing positively, it will incur definite risk to the adequacy in the blood supply and hence endanger patients’ care.

Blood service should also work to retain existing donors to come back for blood donation regularly. Donations from repeated donors are in general regarded as advantageous as they are in general safer and more committed. Besides, regular donors usually have less adverse reaction after donation that make the blood service easier to operate. Therefore, the blood service should develop appropriate strategy in blood donor retention. As such, the donation pattern of the local population should be available in strategies development and their subsequent confirmation.[1–3

**Figure 3:**
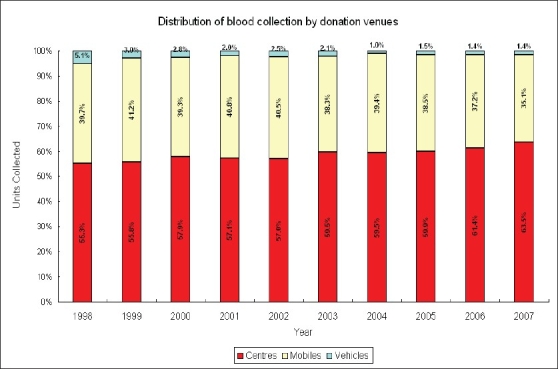
Show the change in the blood collection from mobile (mobiles and vehicles) versus fixed donation venues (centers) in Hong Kong for the past 10 years

**Figure 4:**
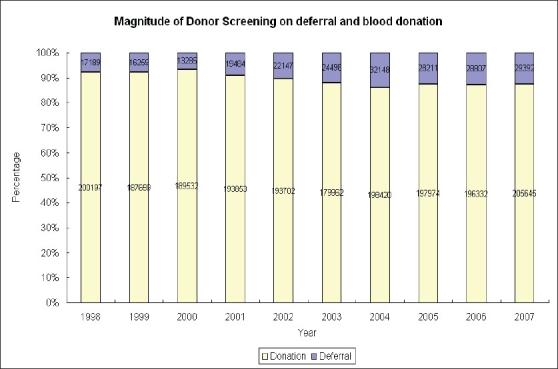
Proportion of deferral in relation to successful blood donation

Lastly, blood service should not routinely use the same format of blood collection. Both intrinsic and extrinsic requirement for blood collection environment are difficult to manage. There is always a problem in coping with donors who are only willing to donate blood at mobile collection sites at the vicinity where they live or work. In other words, it is difficult to mobilize this group of donors to donate blood at donor center. If the blood service is unable to visit these sites, the corresponding donors will gradually lose. The longer the time lapsed from the last donation, the more likely this group of donors will not come back for donation. At the same time, there exist increasing difficulties in organization of the mobile blood collection in developed countries because of the GMP requirement in blood collection in addition to other constraints. For example, parking of mobile donation vehicles in the popular area with high pedestrian flow is almost impossible because of the busy traffic and non-availability of suitable parking space [Figure 3] showed the shrinkage in blood collection from mobile donation vehicles in the last few years. Suitable mobile donation venues that can meet the GMP standards are not easily available. As seen in other developed countries, the BTS has been facing more donor deferral because of increasingly stringent donor eligibility criteria and up to 12% of the prospective donors are deferred from blood donation [Figure 4]. Indeed, donor deferral could have a number of adverse impacts on the blood donation programme such as worsening donor satisfaction arising from discrepancies of understanding between the donors and the BTS on the donor and blood safety.

### Donor recruitment and retention programme in Hong Kong

Since the fundamental principle is to keep the active donor pool healthy and to expand the pool size as far as possible so as to counteract the effects of donor dropout and increasing deferrals from stringent donor eligibility criteria, both new donors and previous donors are the targets of the review. New donors in particular the younger generation are the most important resources and the ideal target in the blood donation programme. As they are usually healthier and able to donate more regularly if committed, therefore, it should be recruited actively into the donor pool to compensate the effects of donor deferral and drop-out. They should be motivated to become a regular blood donor as far as possible. At the same time, those who have donated before should be retained in the active donor pool and invited to come for regular blood donation because previous donors are in general safer in term of infectious disease profiles.

**Figure 5:**
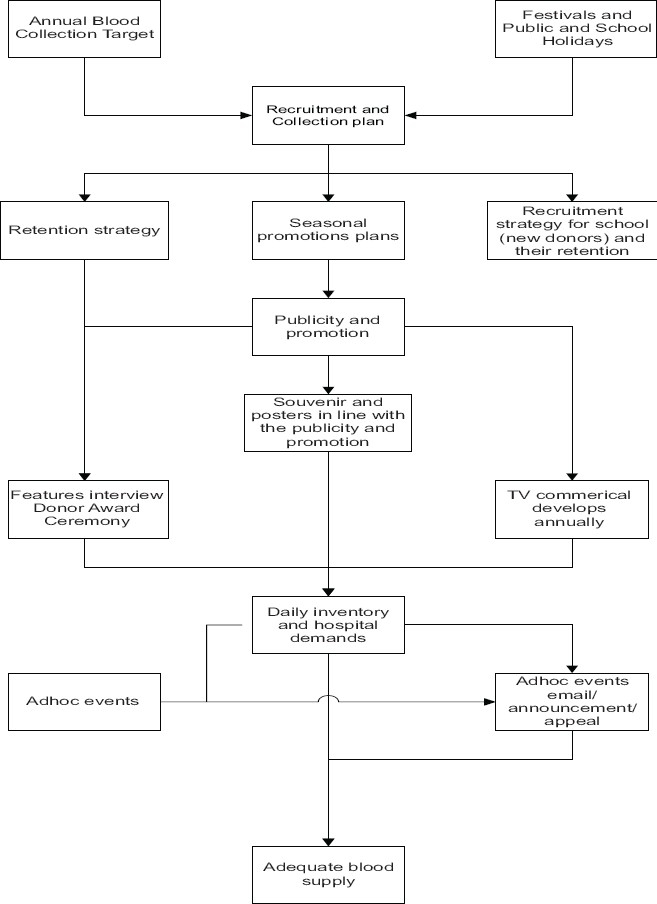
The current approaches in donor recruitment and retention

At present, the BTS has been using combined planned and adhoc approaches for donor recruitment and retention [Figure 5]. Based on the annual blood collection targets, festivals, and public and school holidays, a recruitment and collection plan is prepared. Structural strategies are targeted at donors by promotional publicities and souvenirs presentation at festival periods and selected time, school drives and selected mobile collections. It is coupled with email and radio announcement to encourage and remind them to donate. Whenever there is need for blood of special blood group or a significant surge in blood demand, telemarketing is also used to invite donors to come back. The overall plan has to be adaptive to the local situation, demand and inventory status to avoid any circumstances of insufficiency or over stocking leading to wastage. With this approach, the BTS has been able to maintain adequate blood supply most of the time. But with the ever-increasing demand and problems outlined above, it is getting more common to have occasions when extra efforts have to be made to recall and recruit more donations. Hence, there is a definite need to review and address the current recruitment and retention strategies.

### Future development

Systematic approach on donor recruitment and retention should be developed that includes (1) analysis and study of donation behavior, donor attitude and donor deferral and drop-out;[1,2,4–6] (2) implement of target retention programmes with the analysis of the responses and effectiveness; and (3) monitor the active donor pools and eligible donors of population with reference to the international and local changes in the socio-economic environment and the safety requirement. We have a strong belief that retention should preferably be started at the time of recruitment. Potential donors that once are targeted, should be motivated to donate and develop a sense of good feeling towards the BTS. They should develop their commitment either intrinsically or through extrinsic reminders and/or education. First time donors should be followed-up to check if they will donate again. Reminder can be sent to this group of donors to invite them to donate if the interval is lapsed.[1] Previous donors should follow the same approach to ensure they are retained in the donor pool and continue to donate. As such the previous way of one-off recruitment campaign should be revised to include the above elements.

We propose the following strategies to be employed in donor retention:

1. Development of public awareness and commitment to donate with resources put on recruitment and education focusing on the need of blood donation and the importance or regular donation. We plan to expand the current school programme to further enhance youngster's understanding of the need.
2. Newly recruited donors should be given opportunity to be inspired about the need of regular blood donation even in their first donation. Donors should be attracted to come for donation at a regular base by developing either a donation habit or a retention programme that automatically generate reminders for them to come back. It is not uncommon to see in the feedback that donors may lose their interest or become not satisfied if they feel ignored.
3. Donors should be invited at a predefined interval from the last donation. Hence, resources have to be put in for the understanding the donation pattern..
4. “Important” message should be delivered to all donors on the need of regular blood donation. This should appear regularly and prominently in different communication channels between BTS and the donors. Besides, consideration has to be made to incorporate ways to effective communication with the blood donors. These include the use of mass media, SMS and various internet resources.
5. Donor recognition is an important element to revisit and enhance. All along, donors may be presented with small souvenirs and donor card at the end of donation. But consideration should be made in their design such that the souvenirs might be redesigned as series of collectable items so as to increase their willingness to come back; and holding a blood donor card can be viewed as “prestigious” that attracts the public to join blood donation programme. At the same time, regular donors should be recognized and honored for their long-term commitment by holding donor award ceremony annually.
6. Development of donor activities such as loyalty club for blood donor which can arrange regular recreational activities, social gathering and of course, there should be a measure of the effectiveness to regularly review the pool of regular donors and assess their drop-out rate; and at the same time to be able to develop to monitor the effectiveness of various retention strategies.
7. Last but not the least, donor service and satisfaction should be further improved so that they are willing to come back. Issues like venepuncture skill and follow-up of donation related complications should not be overlooked. Every bit works and contributes to the success of the blood programme and hence a stable and adequate blood supply.

## Conclusion

In summary, both donor recruitment and retention is not an easy task to undergo. It requires dedicate and committed staff, who are willing to understand the problem and explore every possible strategy. Besides, resources and research should be invested in these areas to ensure healthy development.
